# Formic acid-sulfide synergistic aging kinetics and nanometer titanium dioxide modification mechanism in oil-immersed transformer insulation system

**DOI:** 10.1371/journal.pone.0339773

**Published:** 2026-01-23

**Authors:** Yuedong Gao, Zhangcheng Li, Peng Cheng, Yue Zhang, Xiao Wei, Pengfei Jia

**Affiliations:** 1 School of Electrical Engineering, Guangxi University, Nanning, China; 2 TBEA Hengyang Transformer Company Limited, Hengyang, China; 3 School of Chemistry and Chemical Engineering, Guangxi University, Nanning, China; National Chung Cheng University, Taiwan & Australian Center for Sustainable Development Research and Innovation (ACSDRI), AUSTRALIA

## Abstract

This study investigates the synergistic aging effects of formic acid and sulfides on oil-immersed transformer insulation systems and explores the mitigating role of titanium dioxide (TiO₂) nanoparticles. A series of multi-factor coupled accelerated aging experiments were conducted under controlled ambient conditions (25 ± 3 °C) to monitor the mechanical strength of insulating paper and the dielectric breakdown voltage of insulating oil. Results show that formic acid is the dominant agent in early-stage degradation, causing a sharp 63.88% drop in tensile strength within 30 days due to catalytic hydrolysis, followed by partial recovery to 50% of the initial value through crosslinking of degradation products. Notably, the coexistence of formic acid and powdered sulfur causes severe synergistic corrosion, leading to an 82.3% strength reduction and a dramatic decline in breakdown voltage to 7.2 kV. Conversely, the formic acid–DBDS system exhibits a protective synergy, with tensile strength and breakdown voltage recovering to 206 kN/m and 30.2 kV, respectively. The introduction of nano-TiO₂ enhances these effects, especially in DBDS environments, by forming passivation layers and interfacial traps that suppress degradation. These findings provide insights into the nonlinear coupling of chemical corrosion, interfacial modification, and insulation performance evolution in transformer systems.

## 1. Introduction

Oil-immersed transformers are critical components in power systems, and their insu-lation systems are subjected to long-term electro-thermal-mechanical multi-stress coupling effects [1,2]. Among these, the degradation of the mechanical properties of insulating pa-per is one of the primary causes of transformer failure [3–5]. Studies have shown that low-molecular-weight acids generated during thermal aging can catalyze the hydrolysis of cellulose chains in insulating paper. This process reduces the degree of polymerization and leads to a significant loss of mechanical strength [6,7]. In recent years, however, research has revealed that corrosive sulfides react with copper conductors to form copper sulfide deposits, which can further accelerate the degradation of insulating paper [8], presenting new chal-lenges for insulation aging studies [9].

Most existing studies focus on the influence of single aging factors. Cong et al. [10] investigated the mechanism by which copper sulfide deposits affect partial discharge in oil-paper insulation. They found that positive voltage polarity promotes stronger charge accumulation on the copper deposit surface, while negative polarity is more likely to trig-ger reverse discharges at the electrode, highlighting the role of voltage polarity in dis-charge behavior. However, their study only considered the electric field as a single influ-encing factor. Maina et al. [11] examined the behavior of corrosive sulfur in transformer mineral oil and its degradation mechanism for copper conductors and insulating paper. They discovered that corrosive sulfur reacts with copper to form conductive copper sulfide, which deposits on conductor surfaces and adjacent insulation paper, increasing dielectric loss and leading to thermal breakdown failures. Although their work revealed the dielec-tric degradation caused by dibenzyl disulfide (DBDS), a sulfur corrosion by-product, their accelerated aging experiments controlled only the sulfur concentration as a single variable.

Current insulation enhancement technologies mainly focus on the application of nanofluids. Metal oxide nanoparticles such as TiO₂ and Al₂O₃ have been shown to in-crease the breakdown strength of insulating oil by 30%–50% [12–19]. Lv et al. [20] studied the polarity-dependent effects of TiO₂ nanoparticles on streamer propagation and break-down strength in transformer oil under lightning impulse voltage. They found that nano-particles trap fast electrons in shallow traps to form slow electrons, significantly altering the space charge distribution of both positive and negative streamers. However, related studies often neglect the impact of nanoparticles on solid–liquid interfacial properties, particularly their potential role in influencing the mechanical properties of insulating pa-per. Siddique et al. [21] investigated the aging behavior of transformer pressboards im-pregnated with soybean oil-based hybrid insulating liquids containing conductive and semiconductive nanoparticles. They found that nanofluid impregnation significantly im-proved both the mechanical and dielectric properties of pressboards. However, their eval-uation of mechanical degradation was limited to physical indicators such as crystallinity and fiber width, without analyzing the generation of chemical degradation products like low-molecular-weight acids and ketones during aging. These acidic compounds can cata-lyze cellulose chain scission and accelerate hydrogen bond network destruction, while nanoparticles may alter the aging pathway through adsorption effects—an unexplored chemical process [22–29].

In this study, a multi-factor coupled accelerated aging experiment was conducted at room temperature on oil-immersed insulating paper samples under the synergistic influ-ence of formic acid and sulfides. An electronic tensile tester and electrical breakdown ap-paratus were used to study the evolution of mechanical and dielectric properties. The mechanical strength of oil-impregnated paperboard, dielectric strength of aged insulating oil, and dielectric strength of nano-modified insulating oil were tested to reveal the influence of multi-factor coupling on the oil-paper insulation system. Based on changes in tensile strength and the Weibull distribution function, the insulation characteristics of oil-paper at different aging stages were analyzed. Subsequently, a multidimensional performance degradation model was established incorporating both mechanical strength and break-down field strength via Weibull distribution. Experimental measurements of the tensile strength and breakdown voltage (BD) of nano/micro TiO₂-modified insulating paper were conducted. Based on these data, the nonlinear coupling relationships among the concen-tration gradient of chemical corrosion products, interfacial modification effects of nano-particles, and the electromechanical property degradation of insulating paper were sys-tematically analyzed.

## 2. Experimental arrangement

In this section, the experimental methodology is primarily aimed at investigating the impact of aging products in insulating oil on the mechanical strength of insulating pa-per—specifically, examining how formic acid, sulfur elements, and DBDS, either individ-ually or in various combinations, affect the mechanical strength of insulating paper. By measuring the strength of insulating paper samples over four monthly intervals under different aging environments, the corrosiveness of each aging product toward the insulat-ing paper was evaluated. The corrosive effects of formic acid, sulfur, and DBDS were ana-lyzed and compared individually. Additionally, mixtures of modified insulating oil con-taining TiO₂ particles of different sizes with either sublimed sulfur powder or DBDS under formic acid conditions were prepared to study potential synergistic corrosive effects of different sulfur compounds on insulating paper. During the experiments, the pow-er-frequency breakdown voltage of insulating oil under various aging conditions was also compared.

The mechanical strength of the insulating paper was tested using a microcomput-er-controlled universal testing machine (Model WDW-10, Baoding Chaoren Electronic Co., Ltd., China), and the power-frequency breakdown voltage of the insulating oil was meas-ured using a dielectric strength tester (Model HTZIJJ-B, Yangzhou Gaoce Electric Power Equipment Co., Ltd., China).

**Fig 1:**
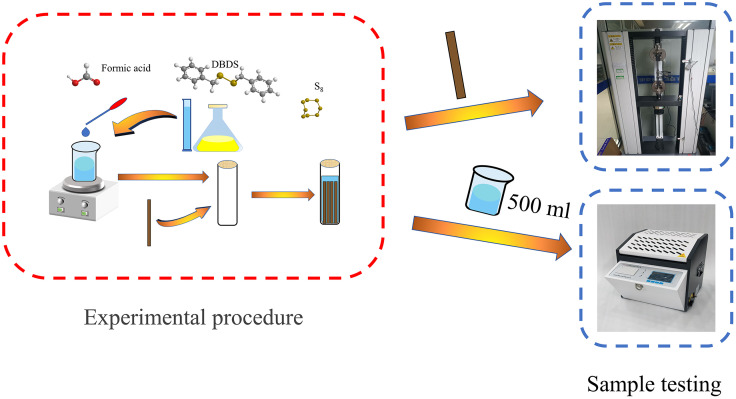
Experimental flowchart.

The process for preparing the corrosion samples and conducting sample testing was shown in Fig 1. Materials and consumables used in the experiment were listed in Table 1. The insulating paperboard used in this study was manufactured by Hunan Guangxin Technology Co., Ltd., with a length of 250 mm, a width of 15 mm, and a thickness of 4 mm. Kunlun No. 25 transformer oil was used for the experiments.

**Table 1:** Consumables used in the experiment.

Experimental Material	Type	Manufacturer
Insulating Oil	KI25X	China KunLun
Insulating Paper Strip	250 × 15 × 4 mm	China GuangXin Technology
Glass Bottle	770 ml	China ZhengQian Glass
Sublimation Powder Sulfur	500 g	China Petroleum and Chemical Corporation
Formic Acid	500 ml	China LinShi Metallurgy
DBDS	25 g	China RunYou Chemical Company
TiO_2_	2 micron	China Zhu Yan Company
TiO_2_	20 nm	Analytical Grade

To simulate the effects of aging products in oil-immersed transformers on the mechanical strength of insulating paper, sealed cylindrical glass jars (bottom diameter: 65 mm; height: 220 mm; capacity: 700 mL) were used as test containers. Rectangular paper strips (250 mm × 15 mm × 4 mm) and transformer oil were separately dried in a vacuum chamber at 80 °C for 48 hours. Then, the paper strips were placed into aging oil that had been pre-treated according to the groupings shown in Tables 2 and 3. Four strips of insu-lating paper were placed in each jar. The groupings were detailed in Table 2: Groups A, B, and C were designed to study the effects of individual aging substances on the tensile strength of insulating paper, while Groups D, E, and F aimed to investigate the effects of combined aging products on tensile strength [30,31].

**Table 2:** Additive combinations.

Experimental Model	Time			
	30 days	60 days	90 days	120 days
Formic Acid (15 ml/L)	A1	A2	A3	A4
Sulfur (0.5 g/L)	B1	B2	B3	B4
DBDS (0.5 g/L)	C1	C2	C3	C4
Formic Acid (15 ml/L) + Sulfur (0.5 g/L)	D1	D2	D3	D4
Formic Acid (15 ml) + DBDS (0.5 g/L)	E1	E2	E3	E4
Formic Acid (15 ml/L) + Sulfur (0.5 g/L) + DBDS (0.5 g/L)	F1	F2	F3	F4

**Table 3:** Additive combinations of titanium dioxide-doped insulating oil.

Experimental Model	Time			
	30 days	60 days	90 days	120 days
TiO_2_ (2 µm,0.2g/L) Formic Acid (15 ml/L) + DBDS (0.5 g/L)	G1	G2	G3	G4
TiO_2_ (2 µm,0.2g/L) Formic Acid (15 ml/L) + Sulfur (0.5 g/L)	H1	H2	H3	H4
TiO_2_ (20 nm,0.2g/L) Formic Acid (15 ml/L) + DBDS (0.5 g/L)	I1	I2	I3	I4
TiO_2_ (20 nm,0.2g/L) Formic Acid (15 ml/L) + Sulfur (0.5 g/L)	J1	J2	J3	J4

Subsequently, to enhance the dielectric breakdown strength of the insulating oil and indirectly mitigate the mechanical degradation of the insulating paper caused by aging products, nano- and micro-sized TiO₂ particles were added to the insulating oil. Table 3 outlines the specific groupings used to explore the influence of different TiO₂ particle sizes in the oil on the tensile strength of the insulating paper.

To ensure that the insulating oil completely immersed the paper strips, each jar was wrapped at the midpoint with plastic wrap after sample preparation, fully covering the lid and secured with tape to minimize the influence of external air and moisture on the oil and experimental results. One paper strip was removed from each jar after one month; thereafter, a strip was removed monthly until all were tested. The tensile strength of the paper strips was measured using a tensile testing machine, which determined the force required to break each sample.

The breakdown voltages of four types of insulating oil were measured and compared with those of the six previously tested aging product conditions. After all paper strips were removed from the jars, the power-frequency breakdown voltage of each oil sample was measured in accordance with the GB/T 507–2002 standard using parallel plate elec-trodes (25 mm diameter, 4 mm thickness), with a voltage ramp rate of 2 kV/s, and six re-peated measurements were conducted per sample.

## 3. Results

### 3.1. Tensile strength test results

In this section, the maximum tensile strength of the samples was measured in accordance with GB/T 12914−2018. A microcomputer-controlled electronic universal testing machine was used to stretch the samples at a constant speed of 20 mm/min until failure, and the tensile strength was determined according to Equation (1)


$$S=\frac{\overset{-}{F}}{b}$$ (1)

**Fig 2:**
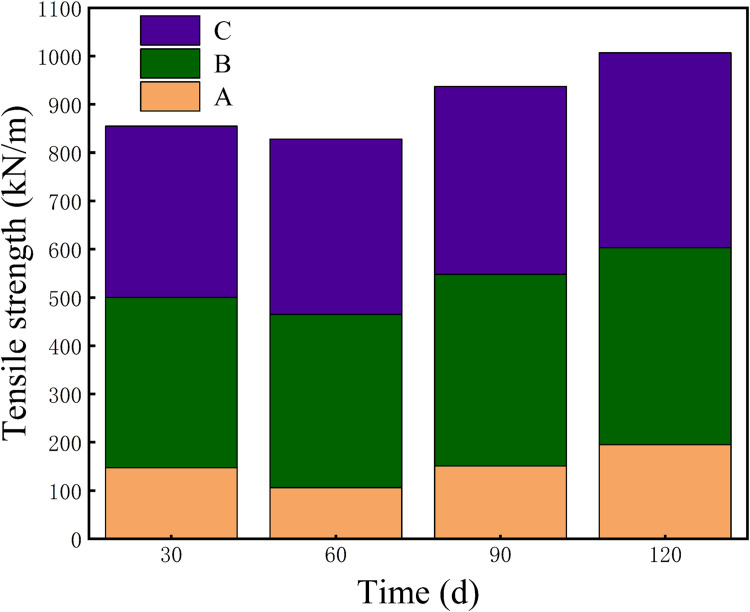
Changes in the effect of single additive combinations on the tensile strength of insulating paper with aging.

The measured tensile force was used to calculate the tensile strength data required for plotting. $\overset{-}{F}$ represents the average maximum tensile force, in newtons (N), and b is the width of the sample, in millimeters (mm). The tensile strength S is expressed in kilonewtons per meter (kN/m).The effect of individual aging products on the tensile strength of insulating paper immersed in insulating oil was shown in Fig 2. The horizontal axis represents the changes in mechanical properties of the paper strips at 30-day intervals.

**Fig 3:**
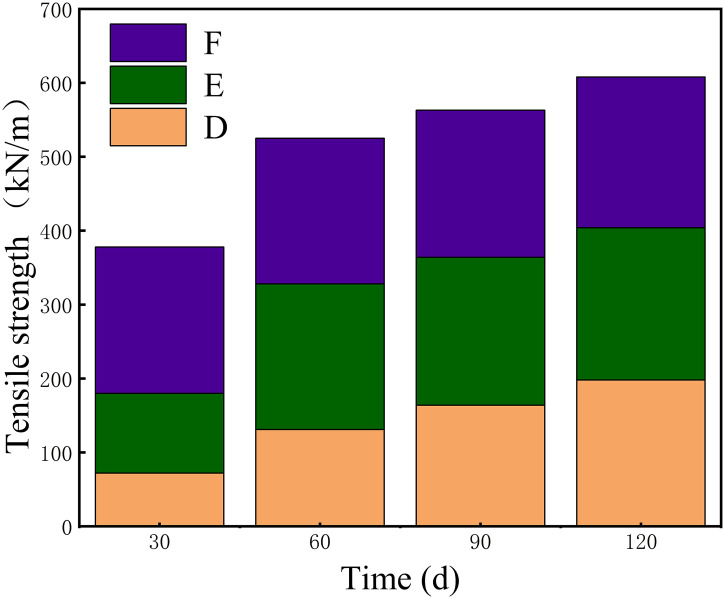
Changes in the effect of different additive combinations on the tensile strength of insulating paper with aging.

In this study, the initial tensile strength of the dried, unimpregnated insulation paper strip was 407 kN/m. Fig 2 presents the evolution of its mechanical properties during thermal aging in insulating oils with different additive combinations. In Group A, where formic acid was present, the tensile strength dropped sharply to 106 kN/m within 60 days. This rapid decline is consistent with the well-known acid-catalyzed hydrolysis of cellulose, which breaks glycosidic bonds and accelerates chain scission in the early stage of aging. A subsequent partial recovery to 202 kN/m at 120 days likely reflects secondary crosslinking among degradation products and the reorganization of the cellulose hydrogen-bond network. In Group B, the early-stage strength only slightly decreased to 353 kN/m, followed by a steady increase to 408 kN/m at 120 days. This behavior suggests that powdered sulfur has limited reactivity toward cellulose under copper-free conditions, allowing the paper structure to stabilize and partially recover as aging progresses. Group C exhibited a similar trend: the strength initially declined to 355 kN/m but gradually rose to 405 kN/m, indicating that DBDS produces milder chemical effects in the absence of catalytic metal surfaces. Overall, the mechanical evolution curves in Fig 2 show that the presence and type of aging products play a decisive role in determining the degradation pathways of cellulose. Formic acid induces strong early-stage hydrolysis, while sulfur-containing substances lead to weaker degradation and allow more pronounced recovery, highlighting the different chemical mechanisms that govern paper aging in transformer insulation systems. Fig 3 shows the effect of different additive combinations on the tensile strength of the insulation paper, with Groups D, E, and F all showing distinct trends. In Group D, the tensile strength dropped sharply to 72 kN/m due to severe early-stage degradation. However, over time, a noticeable recovery occurred, and the strength increased to 199 kN/m at 120 days. Group E experienced a decline to 108 kN/m, followed by a moderate recovery to 206 kN/m, suggesting limited improvement during aging. In Group F, the strength decreased slightly to 198 kN/m and then remained relatively stable throughout the aging process. These results indicate that different additive combinations influence the degradation and recovery behavior of the insulation paper in varying de-grees, likely due to their differing chemical reactivities and interactions during aging. The observed mechanical performance reflects a balance between degradation and stabiliza-tion effects induced by the additives.

Fig 3 illustrates how different additive combinations influence the tensile strength of the insulation paper and the underlying degradation mechanisms during aging. In Group D, the tensile strength dropped sharply to 72 kN/m, reflecting severe early-stage degradation driven by the strong hydrolytic and oxidative effects of the combined aging substances; however, partial recovery to 199 kN/m at 120 days suggests the formation of secondary crosslinking structures and gradual stabilization of the cellulose network. Group E showed a decline to 108 kN/m followed by a moderate recovery to 206 kN/m, indicating that although degradation occurred, the chemical reactivity of the additives was less aggressive and allowed limited structural reorganization. In Group F, the tensile strength decreased slightly to 198 kN/m and then remained relatively stable, implying that the corresponding additives induced milder chemical interactions with cellulose and did not substantially accelerate degradation. Overall, the different trends reflect how each additive combination alters the balance between cellulose chain scission and structural stabilization, highlighting the distinct degradation pathways and recovery mechanisms present in oil-paper insulation systems.

**Fig 4:**
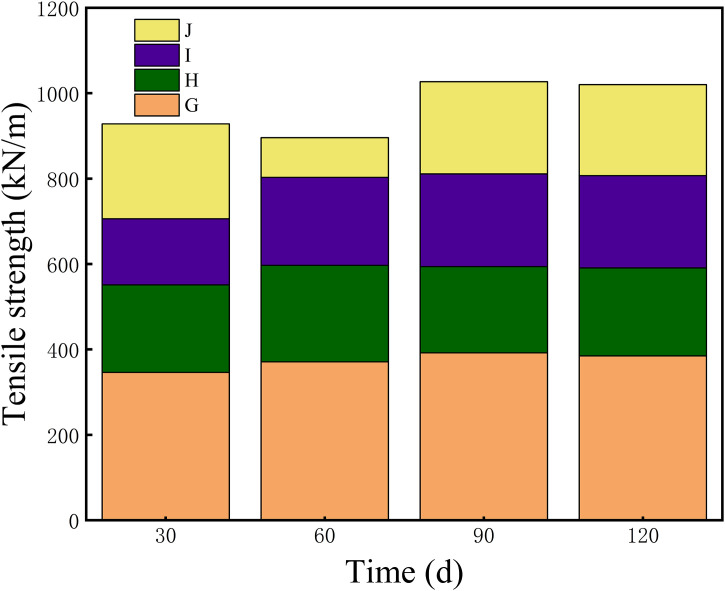
Evolution of the tensile strength of insulating paper during aging in titanium dioxide–doped insulating oil under different additive combinations. The stacked colors represent the contributions of each treatment group: G (TiO₂ only), H (TiO₂ + formic acid), I (TiO₂ + DBDS), and J (TiO₂ + powdered sulfur). Color coding and the expanded legend highlight differences among the chemical environments, enabling clearer comparison of aging behaviors over 30, 60, 90, and 120 days.

Fig 4 illustrates the evolution of the tensile strength of insulating paper in the formic acid–sulfur/DBDS aging environment when assisted by TiO₂–modified insulating oil. The comparison among Groups G, H, I, and J highlights how different chemical conditions influence the protective effect of TiO₂. In Group G, the tensile strength first decreased to 346 kN/m and then partially recovered to 392 kN/m before showing a slight decline at 120 days, indicating a temporary stabilization effect. In Group H, the strength rapidly dropped to 205 kN/m and remained nearly unchanged thereafter, suggesting that formic acid dominates the early-stage degradation. Group I showed an initial decline to 155 kN/m followed by gradual recovery to 216 kN/m, reflecting the milder reactivity of DBDS and the beneficial influence of TiO₂ in this environment. In Group J, the strength continued to decrease throughout aging and reached 213 kN/m, consistent with more severe synergistic degradation. Overall, the trends demonstrate that the effectiveness of TiO₂ is strongly dependent on the surrounding chemical environment, with the most positive effect observed in the DBDS-containing system.

### 3.2. Breakdown voltage test results

During accelerated thermal aging, the hydrolytic cleavage and oxidative degradation of cellulose chains in insulating paper result in the continuous release of low-molecular-weight oxidized cellulose, which migrates into the insulating oil phase. This process introduces soluble polar impurities that significantly impact the dielectric performance of the oil. This study systematically investigates the synergistic effects of ten typical aging environments on this phenomenon. Groups A–F focus on how individual or combined chemical additives influence cellulose dissolution characteristics. Groups G–J further introduce micron-/nano-sized titanium dioxide (TiO₂) to explore how its interfacial behavior modifies the distribution of impurity components. Insulating oil samples from each group were extracted at the 120-day aging endpoint and subjected to power frequen-cy breakdown voltage tests. The results were visualized using both bar charts and Weibull distribution plots. This dual-mode presentation aims to quantitatively correlate the con-centration of polar cellulose-derived impurities with breakdown strength, elucidate how different aging systems differentially affect the dielectric activity of these impurities, and analyze the consistency of breakdown pathways as reflected by the Weibull parameters.

**Fig 5:**
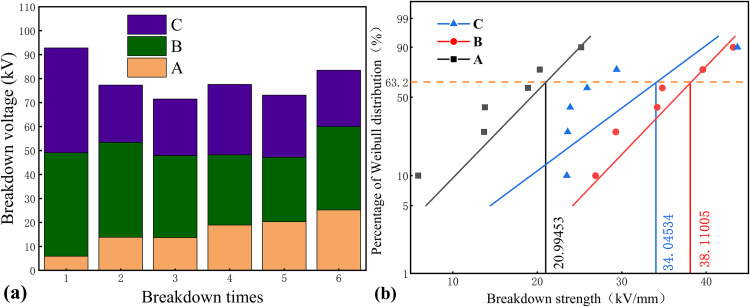
Effect of single additives on breakdown strength of insulating oil. (a) Variation of breakdown voltage with aging and (b) Weibull distribution.

Fig 5 shows the variation in power-frequency breakdown voltage for three oil-paper insulation systems (Groups A, B, and C) under closed room temperature condi-tions. In Group A, the breakdown voltage initially dropped to 5.9 kV but gradually in-creased to 25.2 kV over time, indicating a degree of system stabilization during aging. The corresponding Weibull characteristic value suggests partial recovery of insulation per-formance. In Group B, the voltage decreased from 43.2 kV to a lower point in the early stage, then rose again to 34.8 kV. The Weibull characteristic value of 38.11 kV indicates moderate degradation over time. In Group C, the breakdown voltage dropped sharply at first, showed a brief recovery, and then declined steadily. This reflects a continuous weakening of dielectric strength throughout the aging process. Overall, the results demon-strate that different additive combinations lead to distinct breakdown voltage behaviors. While the formic acid system shows some recovery, systems containing sulfur-based addi-tives tend to exhibit a longer-term decline in breakdown performance.

**Fig 6:**
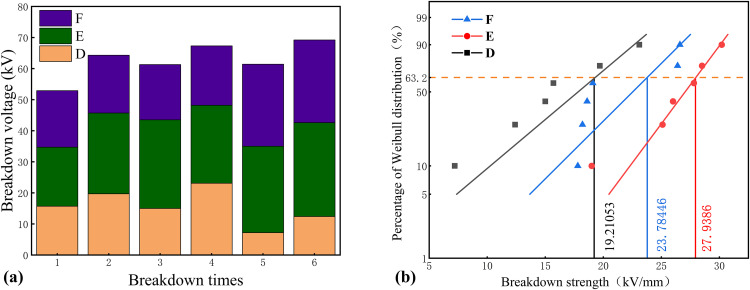
Effect of different additive combinations on breakdown strength of insulating oil. (a) Variation of breakdown voltage with aging and (b) Weibull distribution.

Fig 6 shows the variation of power-frequency breakdown voltage during the aging process for three oil-paper insulation systems with composite additives (Groups D, E, and F). In Group D, the breakdown voltage fluctuated between 15.7 kV and 23.1 kV initially, then dropped sharply to 7.2 kV. Although it later recovered to 12.4 kV, the overall trend showed significant degradation. The Weibull characteristic value was 20.99 kV, and the final voltage decreased by 79% compared to the initial value. In Group E, the breakdown voltage first decreased to 19 kV but gradually increased to 30.2 kV in the later stage. The Weibull characteristic value reached 27.94 kV, and the final voltage rose by 58.9%, show-ing improved insulation performance over time. In Group F, the voltage remained low in the early stage but increased significantly in the later stage, reaching 31.1 kV, with a Weibull characteristic value of 23.78 kV. This corresponds to a 46.2% improvement from the initial level. Overall, Group D showed a continuous decline in performance, while Groups E and F exhibited recovery trends in the later aging stages. These results suggest that different additive combinations lead to varying breakdown behaviors, and later-stage stability plays a key role in improving insulation reliability.

**Fig 7:**
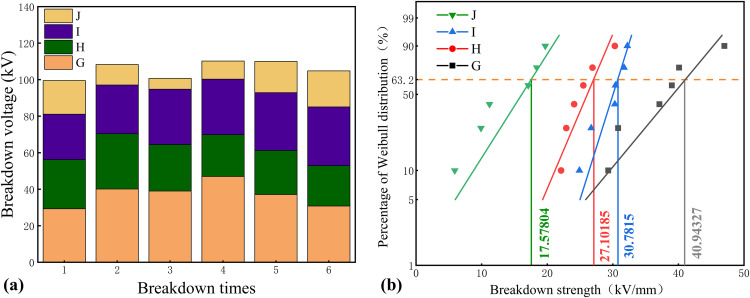
Breakdown performance of insulating oil under different formic acid–sulfur/DBDS composite additive combinations in TiO₂-modified systems. **(a)** Breakdown voltage measured over six repeated tests for each group, with color coding distinguishing the chemical environments: G (TiO₂ only), H (TiO₂ + formic acid), I (TiO₂ + DBDS), and J (TiO₂ + powdered sulfur). The stacked color segments highlight the relative contribution of each treatment to the overall breakdown voltage trend. **(b)** Corresponding Weibull distribution analysis, showing the characteristic breakdown strengths and reliability levels for each group. The clearer legends and color separations facilitate comparison of how TiO₂ interacts with different aging substances to influence dielectric performance.

Fig 7 illustrates the evolution of power-frequency breakdown voltage in oil–paper insulation systems influenced by different combinations of formic acid, sulfur sources, and TiO₂-modified insulating oil. In Group G, the breakdown voltage increased to 47 kV during the early stage but later decreased by 34.5%, indicating that the stabilization effect of TiO₂ diminished with prolonged aging. The Weibull characteristic strength exceeded the final measured value, reflecting a noticeable decline in reliability. Group H exhibited a fluctuating downward trend, reaching a 27.1% reduction at the end of aging; its Weibull characteristic strength was also significantly higher than the final measured voltage, suggesting sustained degradation driven by formic acid. In contrast, Group I showed a gradual and consistent increase in breakdown voltage, ultimately reaching 30.9 kV. The close agreement between the Weibull value and final breakdown voltage indicates enhanced dielectric stability in this environment. Group J showed an initial decrease followed by a moderate recovery, ending at 19.7 kV, with a Weibull characteristic strength of 17.58 kV, reflecting partial improvement under sulfur-rich conditions. Overall, the results demonstrate that the dielectric performance is strongly dependent on the chemical environment, with the DBDS–TiO₂ system showing the most favorable stabilization effect among the tested groups.

## 4. Discussion

**Fig 8:**
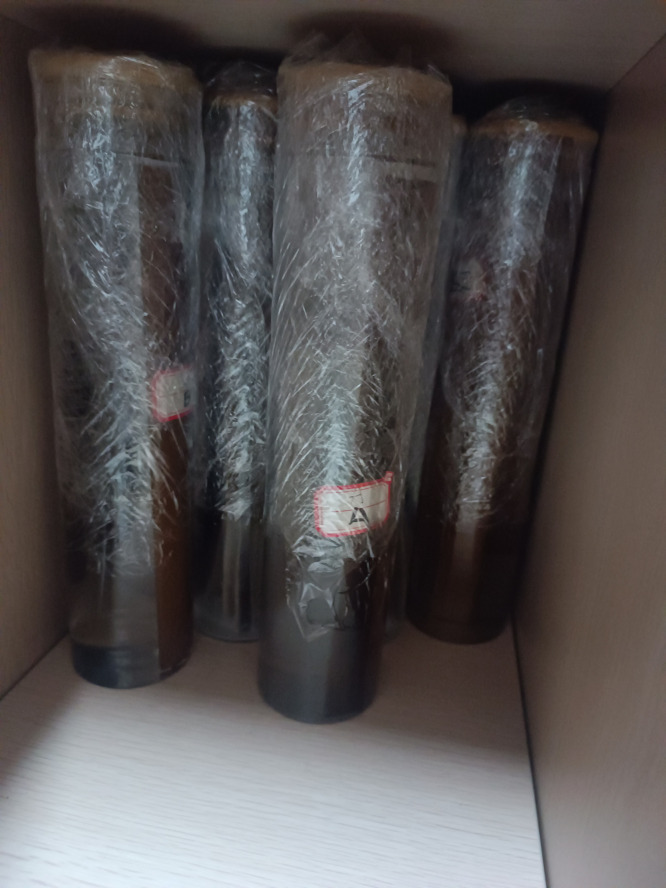
Simulate the effects of aging products in oil-immersed transformers.

As shown in Fig 8, the mechanical and dielectric properties of the oil–paper insulation system exhibit coupled evolution under different aging products. Formic acid–based systems showed rapid early-stage deterioration, driven mainly by acid-catalyzed hydrolysis of cellulose chains and the accelerated formation of polar degradation products in the oil. Although partial recovery occurred in the later stage, the underlying chemical pathways indicate that formic acid remains the dominant driver of severe initial degradation.

In contrast, sulfur-containing systems demonstrated milder early-stage deterioration. Both powdered sulfur and DBDS gradually allowed stabilization of the oil–paper structure as aging progressed. The weaker corrosive activity of these sulfur species—especially in the absence of catalytic copper—enabled partial re-organization of the cellulose hydrogen-bond network, resulting in improved tensile behavior and more stable dielectric response in the later stage. DBDS-based systems exhibited the most consistent stabilization trend, suggesting that DBDS undergoes slower decomposition and generates less aggressive intermediates under the tested conditions.

When formic acid coexisted with sulfur species, the system behavior changed significantly. The formic acid–powdered sulfur combination produced a highly aggressive aging environment, reflecting strong synergistic interactions between acidic and elemental sulfur pathways. Only limited recovery was observed, indicating persistent chemical instability. In contrast, the formic acid–DBDS combination showed more moderate behavior, implying that DBDS-derived intermediates may partially buffer or slow the acid-driven degradation process, leading to more controlled aging dynamics.

The performance of TiO₂ was closely related to both particle size and chemical environment. Micron-scale TiO₂ provided an initial improvement in dielectric strength but failed to maintain stability during prolonged aging, likely due to weaker dispersion and reduced interfacial activity. Nano-TiO₂ showed more consistent protective behavior, especially in DBDS-containing systems, where its high surface area enhanced adsorption of polar species and contributed to improved long-term stability. However, in sulfur-rich systems, nano-TiO₂ displayed reduced effectiveness, indicating possible incompatibility with the reaction pathways associated with elemental sulfur.

Overall, the discussion reveals that early degradation is dominated by the chemical reactivity of aging substances—primarily formic acid—while later recovery is influenced by structural stabilization, crosslinking, and interfacial interactions. Nano-TiO₂ offers meaningful improvement only in environments with moderate chemical reactivity, such as DBDS systems. Therefore, practical applications should tailor additive strategies to the specific sulfur source present in the insulation oil. Controlling formic acid concentrations and optimizing maintenance schedules for insulation paper may further enhance long-term reliability.

## 5. Conclusion

This study systematically investigates the aging mechanisms of insulating paper in different aging products and elucidates the reasons for these changes from the perspec-tives of mechanical strength variations and breakdown voltage variations of the insulating oil used. The conclusions are as follows:

(1) Formic acid is a key factor in the early-stage mechanical degradation of insulation paper. It causes a sharp decline in tensile strength at the beginning of aging. In the later stage, partial strength recovery was observed, resulting in a “V”-shaped trend during the aging process.
(2) Different sulfur compounds lead to different aging behaviors. Powdered sulfur helps improve mechanical strength in the long term, while DBDS shows moderate protective effects. However, when combined with formic acid, both compounds accelerate deg-radation, leading to more severe performance decline.
(3) Nano-TiO₂ performs well in DBDS-containing systems at the experimental concentration used in this study (0.2 g/L), improving both tensile strength and breakdown voltage over time. In contrast, its effectiveness is reduced in systems containing powdered sulfur, where performance deterioration was more pronounced. Therefore, under the tested concentration of 0.2 g/L, nano-TiO₂ is more suitable for DBDS-based aging environments, whereas alternative modification strategies may be required for sulfur-rich systems.

This study systematically reveals the synergistic degradation mechanisms of formic acid and sulfur compounds during the aging of transformer oil-paper insulation systems. It clarifies the distinct impact pathways of different sulfur sources on the structural integ-rity of insulation paper and the dielectric performance of oil. Furthermore, it confirms that nano-TiO₂ can significantly enhance system stability in specific systems through a syner-gistic mechanism involving adsorption, passivation, and trap formation. The findings show that the nonlinear evolution of insulation performance results from the dynamic competition between chemical degradation and interfacial self-healing. The effectiveness of nanomodification strategies depends on the type of additives and interfacial reaction characteristics. This work not only deepens the understanding of the multi-factor coupling mechanisms in oil-paper aging but also provides theoretical support and practical guid-ance for material selection and aging suppression in high-reliability transformer insula-tion systems.

## Supporting information

S1 File Minimal data set. This file contains the minimal data set required to replicate the results of this study, including the underlying numerical values used to calculate reported means, standard deviations, and other statistical measures, the raw data used to generate all figures and graphs, and the data points extracted for image-based analyses, together with the associated metadata and methodological descriptions.
(DOCX)

## References

[1] Chen Q, Sun W, Cheng S, Zhang L, Li C. A two-step modification method for estimating the aging status of insulation paper after oil-replacement based on the methanol. IEEE Trans Dielect Electr Insul. 2025;32(2):1129–37. doi: 10.1109/tdei.2024.3515922

[2] Jeanneton A, Perrier C, Beroual A. Thermal aging comparison of alternative liquids for oil-paper high-voltage insulation in current transformers. IEEE Trans Dielect Electr Insul. 2025;32(2):1123–8. doi: 10.1109/tdei.2024.3508612

[3] Huang X, Zhou Y, Gesang Q, Zhang L, Zhang Y, Teng C, et al. Construction of nanocellulose sandwich-structured insulating paper and its enhancement for mechanical and electrical properties. IEEE Trans Dielect Electr Insul. 2021;28(4):1127–35. doi: 10.1109/tdei.2021.009446

[4] Huang J, Zhou Y, Zhang L, Zhou Z, Huang X, Zeng X. Study on the electrical properties of nanopaper made from nanofibrillated cellulose for application in power equipment. Cellulose. 2018;25(6):3449–58. doi: 10.1007/s10570-018-1782-7

[5] Hao J, et al. Structure and space charge inhibition performance of reactive RF magnetron sputter deposited Al 2 O 3 thin film on the fiber surface. 4th Annu. Int. Conf. Mater. Eng. Appl. (ICMEA 2017). 2018.

[6] Singha S, Asano R, Frimpong G, Claiborne CC, Cherry D. Comparative aging characteristics between a high oleic natural ester dielectric liquid and mineral oil. IEEE Trans Dielect Electr Insul. 2014;21(1):149–58. doi: 10.1109/tdei.2013.003713

[7] Lundgaard L, Hansen W, Ingebrigtsen S. Ageing of mineral oil impregnated cellulose by acid catalysis. IEEE Trans Dielect Electr Insul. 2008;15(2):540–6. doi: 10.1109/tdei.2008.4483475

[8] Cong H, Wang Y, Pan H, Hu X, Li Q. Multiple sulfur corrosion simulation on the oil–paper insulation system under synergistic electric and thermal fields. IEEE Trans Dielect Electr Insul. 2023;30(2):634–42. doi: 10.1109/tdei.2022.3216232

[9] Zhang Y, Huang Z, Wang F, Wang Q. Investigation on the microscale interaction characteristics of POSS-modified vegetable insulating oil under nonuniform temperature and electric fields. IEEE Trans Dielect Electr Insul. 2024;31(6):3497–503. doi: 10.1109/tdei.2024.3439496

[10] Cong H, Su W, Wang Y, Qiao L, Li Q, Xu M. Mechanism of copper sulfide deposition on oil-paper insulation partial discharge under extremely uneven electric field. IEEE Trans Dielect Electr Insul. 2025;32(2):1027–35. doi: 10.1109/tdei.2024.3466113

[11] Maina R, Tumiatti V, Pompili M, Bartnikas R. Corrosive sulfur effects in transformer oils and remedial procedures. IEEE Trans Dielect Electr Insul. 2009;16(6):1655–63. doi: 10.1109/tdei.2009.5361586

[12] Zhai X, Zhang D, Li X, Shao X, Zhang T, Zhan J, et al. Enhancement and prediction of mechanical properties of microcellulose/ nanocellulose-modified insulating paper. IEEE Trans Dielect Electr Insul. 2024;31(5):2642–51. doi: 10.1109/tdei.2024.3422159

[13] Masra SMW, Arief YZ, Sahari SK, Muhammad MS, Rigit ARH, Rahman MR. A systematic review on promising development of palm oil and its nanofluid as a biodegradable oil insulation alternative. IEEE Trans Dielect Electr Insul. 2022;29(1):302–18. doi: 10.1109/tdei.2022.3146594

[14] Nor SFM, Azis N, Jasni J, Ab Kadir MZA, Yunus R, Yaakub Z. Investigation on the electrical properties of palm oil and coconut oil based TiO2 nanofluids. IEEE Trans Dielect Electr Insul. 2017;24(6):3432–42. doi: 10.1109/tdei.2017.006295

[15] Sun P, Sima W, Jiang X, Zhang D, He J, Chen Q. Failure of nano-modified oil impregnated paper under repeated impulse voltage: Effects of TiO2 nanoparticles on space charge characteristics. IEEE Trans Dielect Electr Insul. 2018;25(6):2103–11. doi: 10.1109/tdei.2018.007173

[16] Lv Y, Zhou Y, Li C, Ge Y, Qi B. Creeping discharge characteristics of nanofluid-impregnated pressboards under AC Stress. IEEE Trans Plasma Sci. 2016;44(11):2589–93. doi: 10.1109/tps.2016.2597362

[17] Chakraborty B, Maur S, Pradhan AK, Chatterjee B, Dalai S. Insight the impact of TiO2 and Al2O3 nanoparticles in mineral, vegetable oil based on ac dielectric properties—conformity with weibull distribution. IEEE Trans Plasma Sci. 2023;51(10):3095–102. doi: 10.1109/tps.2023.3311910

[18] Huang M, Chen L, Zhang L, Li Y, Niu M. Effect of carrier mobility on corona discharge of transformer oil under DC voltage. IEEE Trans Plasma Sci. 2023;51(12):3593–600. doi: 10.1109/tps.2023.3331081

[19] Shan B, Ying Y, Huang M, Niu M, Qi B, Li C, et al. Effect of TiO2 nanoparticles on DC breakdown performance of transformer oil-impregnated pressboard. IEEE Trans Dielect Electr Insul. 2019;26(6):1998–2004. doi: 10.1109/tdei.2019.008300

[20] Lv Y, Ge Y, Li C, Wang Q, Zhou Y, Qi B, et al. Effect of TiO2nanoparticles on streamer propagation in transformer oil under lightning impulse voltage. IEEE Trans Dielect Electr Insul. 2016;23(4):2110–5. doi: 10.1109/tdei.2016.7556485

[21] Siddique ZB, Basu S, Basak P. Aging of transformer pressboard impregnated with conductive and semi-conductive nanoparticles dispersed soyabean oil blend. IEEE Trans Dielect Electr Insul. 2022;29(6):2340–7. doi: 10.1109/tdei.2022.3201441

[22] Gao S, Lei Y, Liu Y, Huang C, Yang L. The corrosion and activation mechanism of thiophenic sulfides in the oil-paper insulation. IEEE Trans Dielect Electr Insul. 2025;32(3):1353–63. doi: 10.1109/tdei.2025.3539260

[23] Gao L, Chen Y, Lv Z, Zhou J, Wu K. Effect of insulating oil type on double electric layer at oil-paper interface. IEEE Trans Dielect Electr Insul. 2025;32(3):1484–91. doi: 10.1109/tdei.2024.3496613

[24] Chen C, Yang L, Feng S, Zhao W, Wang X, Chen Z, et al. Influence of cellulose particle aggregation on transformer oil dielectric strength. IEEE Trans Dielect Electr Insul. 2025;32(3):1606–14. doi: 10.1109/tdei.2024.3486682

[25] Ye W, Hao J, Gao C, Qiao X, Zhang J, Liao R. Molecular mechanism analysis of multicomponent mixed insulating oil improving lightning impulse discharge characteristics of oil-impregnated paper. IEEE Trans Dielect Electr Insul. 2025;32(3):1615–22. doi: 10.1109/tdei.2024.3486268

[26] Herlin Rose AP, Srinivasan M, Sarathi R, Sarathkumar D. Experimental aging of three-element mixed oil insulation and pressboard for power transformer applications. IEEE Trans Dielect Electr Insul. 2025;32(3):1768–76. doi: 10.1109/tdei.2024.3472630

[27] Mariprasath T, Kishore P, Padmavathi M. Feasibility analysis of new green liquid dielectrics for transformers. Green Materials. 2024;12(1):41–5. doi: 10.1680/jgrma.23.00038

[28] Mariprasath T, Kirubakaran V, Amaresh K, Khan B. Investigation of Pungamia oil properties using spectroscopy for transformer applications. IETE Journal of Research. 2023;70(4):4169–79. doi: 10.1080/03772063.2023.2198990

[29] Mariprasath T, Ravindaran M. An experimental study of partial discharge analysis on environmental friendly insulating oil as alternate insulating material for transformer. Sādhanā. 2022;47(4). doi: 10.1007/s12046-022-01946-8

[30] Li Y, Li R, Yang S, Guo C, Xu Y, Zhou K, et al. DC breakdown characteristics of transformer oil with different cellulose impurity concentrations. IEEE Trans Dielect Electr Insul. 2025;32(3):1810–9. doi: 10.1109/tdei.2024.3488680

[31] Liang K, Wang F, Zhong L, Chen S, Sun Q, Hu C, et al. Investigation into the formation mechanisms of soluble copper ions in oil: Reconsidering the impact of corrosive sulfides and acids. IEEE Trans Dielect Electr Insul. 2024;31(2):683–93. doi: 10.1109/tdei.2024.3370131

