# Supplementary figures and images for: Formic acid-sulfide synergistic aging kinetics and nanometer titanium dioxide modification mechanism in oil-immersed transformer insulation system
Source: PLoS One. 2026 Jan 23;21(1):e0339773. doi: 10.1371/journal.pone.0339773 (PMC12829876; doi:10.1371/journal.pone.0339773)

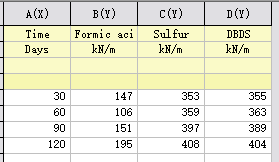


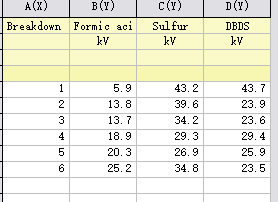


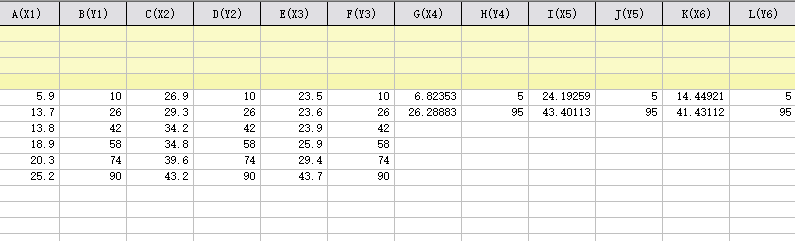


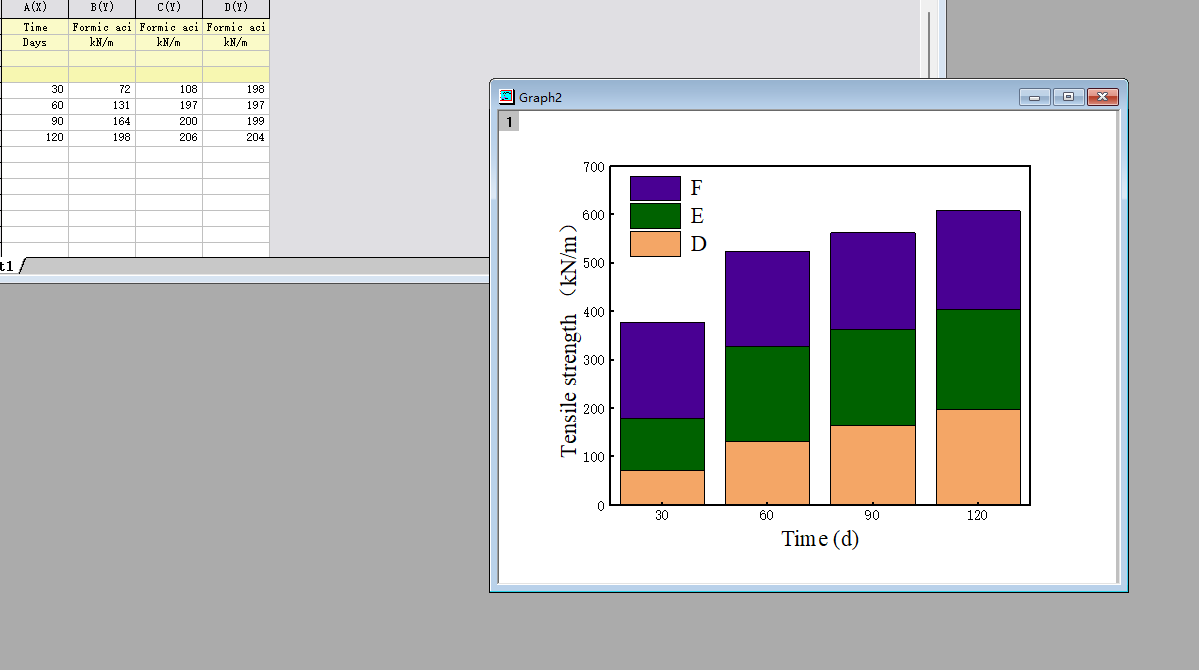


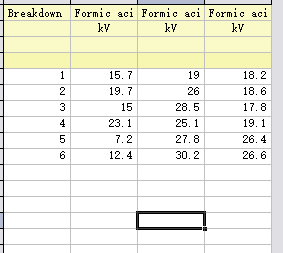


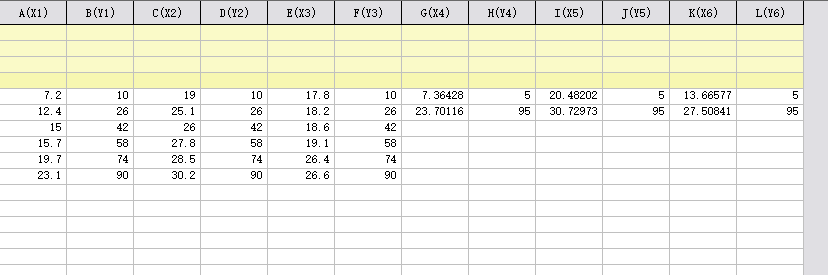


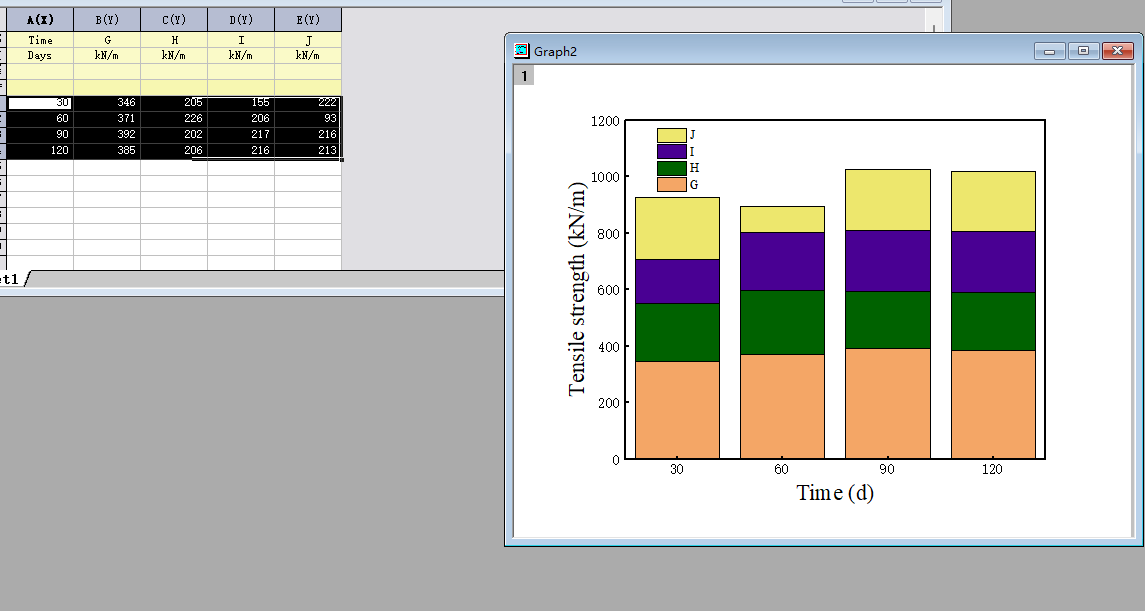


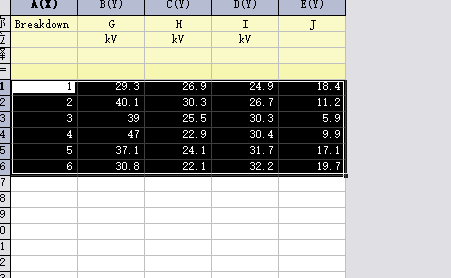


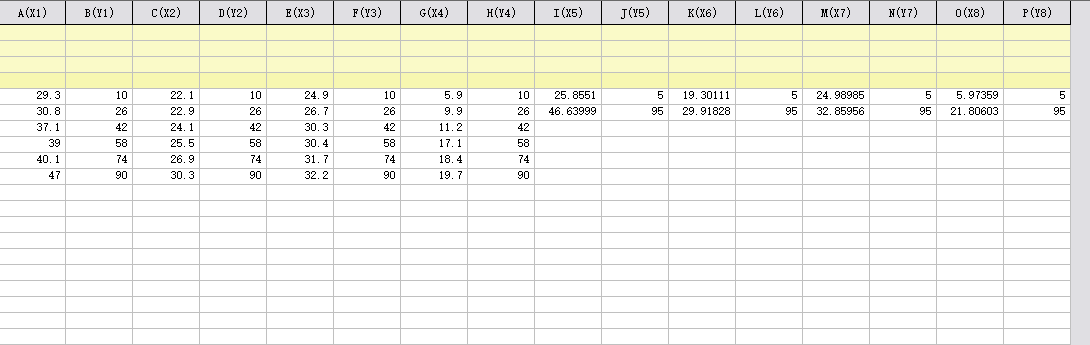

Supplement: S1 File — This file contains the minimal data set required to replicate the results of this study, including the underlying numerical values used to calculate reported means, standard deviations, and other statistical measures, the raw data used to generate all figures and graphs, and the data points extracted for image-based analyses, together with the associated metadata and methodological descriptions. (DOCX) [file pone.0339773.s001.docx]
